# Phytochemical Screening, Phenolic Compounds and Antioxidant Activity of Biomass from *Lychnis flos-cuculi* L. In Vitro Cultures and Intact Plants

**DOI:** 10.3390/plants10020206

**Published:** 2021-01-22

**Authors:** Michał P. Maliński, Małgorzata Anna Kikowska, Agata Soluch, Mariusz Kowalczyk, Anna Stochmal, Barbara Thiem

**Affiliations:** 1 Chair and Department of Pharmaceutical Botany and Plant Biotechnology, Poznan University of Medical Sciences, 14 Św. Marii Magdaleny St., 61-861 Poznań, Poland; kikowska@ump.edu.pl (M.A.K.); bthiem@ump.edu.pl (B.T.); 2 Department of Biochemistry and Crop Quality, Institute of Soil Science and Plant Cultivation, State Research Institute, 8 Czartoryskich St., 24-100 Puławy, Poland; asoluch@iung.pulawy.pl (A.S.); mkowalczyk@iung.pulawy.pl (M.K.); asf@iung.pulawy.pl (A.S.)

**Keywords:** ragged robin, phytochemical screening, in vitro cultures, ferric reducing antioxidant potential, DPPH radical scavenging activity, total phenolics, total flavonoids, total phenolic acids

## Abstract

*Lychnis flos-cuculi* L., a species with potential medicinal value, contains flavonoids, phenolic acids, triterpenoid saponins and ecdysteroids. In this study, the antioxidant activity of plant material of *L. flos-cuculi* obtained from in vitro cultures compared to that of intact plants from the natural site has been evaluated for the first time. Phytochemical screening of the in-vitro-derived material by ultra-high-performance liquid chromatography mass spectrometry (UHPLC-MS) confirmed the presence of the aforementioned metabolite classes. The aqueous methanolic extracts from in-vitro-derived plant material and the organs of intact plants were analyzed using spectrophotometric methods to quantify total phenolics, phenolic acids and flavonoids, and determine the preliminary antioxidant activity by ferric reducing antioxidant potential (FRAP) and DPPH radical scavenging activity assays. The results showed that the inflorescence (Ns-F), and flowering herb of both plants gathered from natural habitat (Ns-H) and in-vitro-derived plants from the experimental plot (ExV-H) are the materials richest in polyphenols (195.4, 113.47, 112.1 mg GAE g^−1^ d.w., respectively), and demonstrate the highest antioxidant activity (20.14, 11.24, and 11.46 mg AAE g^−1^ d.w.). The extract from callus exhibited the lowest polyphenol content and antioxidant potential. The contents of total phenolics, flavonoids and phenolic acids correlate with the results of the antioxidant capacity of *L. flos-cuculi* extracts.

## 1. Introduction

*Lychnis flos-cuculi* L. (*Silene flos-cuculi* (L.) Greuter and Burdet, *Coronaria flos-cuculi* (L.) A. Braun, Ragged Robin) is an herbaceous plant of Caryophyllaceae family, growing on wet meadows and floodplains throughout Europe and Northern Asia. Due to the changes in the traditional cultivation and exploitation of meadows and their drainage to form arable land, the population of this plant is diminishing. The species has been briefly mentioned in traditional medicine as a remedy for migraine and intestinal pain, and as possessing wound-healing properties [1,2,3].

To date, several reports have demonstrated that *L. flos-cuculi* contains phenolic acids, flavonoids, ecdysteroids, and a significant quantity of complex triterpenoid saponins [1,4,5,6,7]. Phenolic acids include simple hydroxy- or methoxy-derivatives of benzoic and *trans*-cinnamic acids, together with caffeoyl-substituted quinic acid derivatives. Flavonoids were found to be C-glycosyl derivatives of apigenin and luteolin, as well as O-glycosides: rutoside and apigenin-7-O-glycoside [1,6,7]. The only triterpenoid saponins described to date are coronosides A and B, glycosides of gypsogenin and hederagenin of only partially elucidated structure [4]. Ecdysteroids, with 20-hydroxyecdysone (20HE) and polypodine B (polB) as major constituents, were reported by Báthori et al. [5] and Mamadalieva et al. [8]. Each class of these metabolites presents an opportunity to utilize their biological activity [9,10,11]. Phenolic acids and flavonoids are antioxidants exerting a whole spectrum of protective effects, discussed below [11]. The activity of triterpenoid saponins varies depending on their structure, including expectorant, immunostimulatory, anti-inflammatory, hypocholesterolemic and anticarcinogenic effects, as well as a spectrum of biocidal effects related to their membrane-permeabilizing action [10]. Ecdysteroids are known for their non-androgenic, mild anabolic activity, wound-healing effects and adaptogenic properties, as well as the modulation of lipid and carbohydrate metabolism [9]. However, knowledge of the presence of secondary metabolites in underground parts of *L. flos-cuculi* and plants propagated in in vitro conditions is fragmentary and incomplete [12].

The antioxidant activity, including the radicalscavenging and redox potential of polyphenols, is generally thought to be a reason behind their beneficial effect on human health. Based on in vitro assays, the effects comprise neuroprotective, cardioprotective, anti-inflammatory, anti-cancer and antibacterial properties [11,13,14,15]. It is now proposed, however, that the dietary intake of some phenolic compounds may actually exert a mild pro-oxidant effect and trigger ROS generation. The result is a hormetic response, inducing the expression of enzymatic antioxidant systems, such as superoxide dismutase, catalase and glutathione. This, in turn, may actually slow the progression of degenerative diseases, affect the process of ageing and prolong the lifespan, as well as modulating the inflammatory responses [16,17]. Still, there is a correlation between the intake of phenolic acids and flavonoids and the prevention of oxidative stress and the progression of degenerative diseases, including cancer, neurodegenerative diseases, cardiovascular diseases, and type II diabetes [11,14,17]. Regardless of the actual mechanism of action, the evaluation of the phenolic content and antioxidant activity of plant extracts is still the common subject of phytochemical analysis. The antioxidant activity and the content of total phenolic compounds of in-vitro-derived *L. flos-cuculi* material, in comparison to natural site plant material, have not been evaluated to date. Underground parts of the plant have not been investigated either.

Plant in vitro cultures can provide a sufficient quantity of uniform biomass of high quality under controlled conditions and affect secondary metabolite production in the medicinal species [13,18,19]. Moreover, this biomass may be a good sample of the plant material for phytochemical and biochemical investigation [13,20,21]. In our previous studies regarding *L. flos-cuculi*, the efficient protocol of rapid micropropagation was described for the first time [12]. There are few research papers on the phytochemical study [4,5,6,7,8] and the biological activity [7,8] of *L. flos-cuculi* extracts, therefore, this work enriches the knowledge on the profile of compounds that are present in micropropagated plants.

The aim of the present study was to determine and compare the content of total phenolics (TP), total phenolic acids (TPA) and total flavonoids (TF) in the aqueous methanolic extracts of diverse biomass obtained from in vitro cultures and wild plants of *L. flos-cuculi*. The preliminary evaluation of the antioxidant activity was performed by means of two assays, relying on two different mechanisms to find a connection between polyphenol accumulation and the antioxidant capacity. In addition, the extracts of callus and the flowering herb and roots of micropropagated plants growing in the experimental plot were subjected to phytochemical screening using the chromatography and mass spectrometry methods. These experiments aimed at the selection of the plant material from the studied species, rich in phenolic compounds and exhibiting high antioxidant activity.

## 2. Results

Polyphenols are known for their biological activity related to their chemical properties as antioxidants [22]. Typically, the amount of polyphenols depends on the stage of development of the plant and is the highest during flowering. The objective of this study was to determine and compare the content of polyphenols in the diverse plant material obtained from in vitro cultures and gathered from the natural habitat.

### 2.1. Plant Material Obtained from In Vitro Cultures

**Figure 1:**
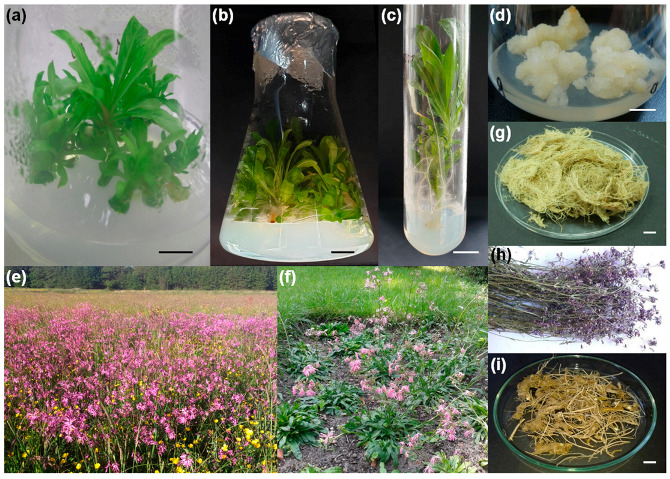
*Lychnis flos-cuculi.* In vitro cultures: (**a**) young shoot culture, (**b**) multiplied shoots, (**c**) rooted shoot, (**d**) hypocotyl-derived callus. Mature plants: (**e**) flowering plants at natural site, (**f**) flowering micropropagated plants on experimental plot (ex vitro). Dried plant material: (**g**) in-vitro-derived roots, (**h**) flowering herb and (**i**) roots of micropropagated plants. The bars shown for scale are 1 cm in width.

Shoots were propagated and rooted under in vitro conditions (Figure 1a–c). Shoots and adventitious roots from the multiplied plantlets were collected for the analysis (Figure 1b,g). Micropropagated plants transferred to the experimental plot (ex vitro plants, Figure 1f) developed the root system and reached flowering and fruition. The flowering herb was gathered along with abundant roots (Figure 1h,i). The plants from the experimental plot were selected for analysis because they are easily obtained due to the high efficacy of micropropagation and genetic uniformity of the material confirmed earlier [12]. They are also more easily harvestable, contrary to wild plants growing on dense, wet meadows, and their root systems are not entangled with those of other species. The flowering stems of micropropagated plants are also more branched and produce more flowers than natural site plants. Hypocotyl-derived callus, demonstrating the high growth rate, was used for the analysis (Figure 1d). Intact plants collected from the natural habitat (Figure 1e) were compared to in vitro biomass.

The plant material used for further assays and analyses included biomass of in vitro shoot cultures (InV-S), in-vitro-regenerated adventitious roots (InV-R), and callus (InV-C). Additionally, the plant material included the flowering herb (ExV-H) and roots of micropropagated (ex vitro) plants (ExV-R), as well as plant parts gathered from the natural site for comparison, that is, inflorescences (Ns-F), the flowering herb (Ns-H) and roots of flowering intact plants (Ns-R).

### 2.2. Preliminary TLC Analysis

**Figure 2:**
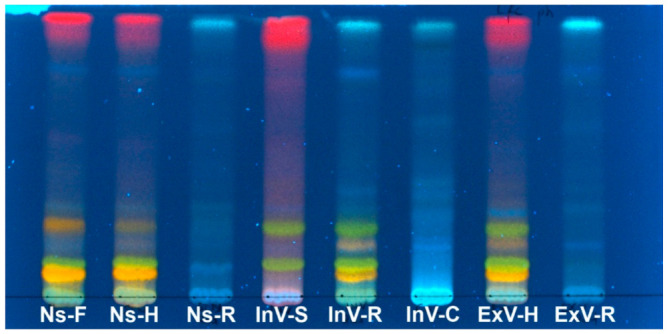
TLC chromatogram of *Lychnis flos-cuculi* 70% hydromethanolic extracts from different organs and sources. Samples: Ns-F—natural site flower, Ns-H—natural site herb, Ns-R—natural site root; InV-S—in vitro shoots, InV-R—in vitro roots, InV-C—callus tissue; ExV-H—ex vitro herb, ExV-R—ex vitro roots. Stationary phase: silica gel. Mobile phase: ethyl acetate-acetic acid-water (8:1:1). Observed under 366 nm UV light after derivatization with 0.1% aminoethyl diphenylborate ethanolic solution.

The extracts of dried plant material obtained from in vitro cultures and intact plants were analyzed by TLC chromatography for initial screening and comparison. After derivatization of the plate with all tested extracts with NA reagent and inspection under UV light, four distinct flavonoid compounds were detected in most samples, except natural site root, ex vitro root and callus samples, where no yellow or orange fluorescence specific to flavonoids was visible (Figure 2). The similar spots suggest the occurrence of the same flavonoid constituents in inflorescence and flowering herbs; the spots from ex vitro herb extract are slightly more intense. The flavonoids accumulated by in-vitro-derived roots are very similar to those present in flowering herb from the experimental plot. Only two of these are present in shoot culture extract. Turquoise spots show the presence of phenolic acids in callus and roots, revealing similarities between natural site and ex vitro roots, as well as callus. The phenolic acid constituents of in-vitro-derived roots additionally resemble those in aerial parts of the plant. Not all compounds revealed in aerial parts are encountered in shoot cultures, perhaps due to their presence in flowers. Closer investigation of callus extract by 2D-TLC and derivatization with AlCl_3_ did not reveal flavonoids (Figure S1, Supplementary Materials). All the samples contained phenolic acids. Flavonoid compounds detected in ex vitro flowering herb showed a very similar qualitative composition when compared to the natural site herb. This was the additional reason for choosing ex vitro plant material for phytochemical screening.

### 2.3. UHPLC-MS Analysis

**Figure 3:**
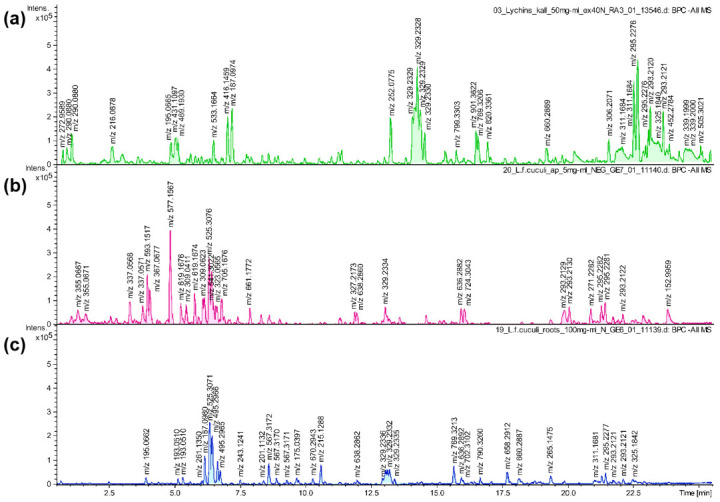
The base-peak chromatograms (BPC) of aqueous methanolic extracts from *Lychnis flos-cuculi* (**a**) callus, (**b**) aerial parts of ex vitro plants, (**c**) roots of ex vitro plants, obtained using high resolution ultra-high-performance liquid chromatography-electrospray ionization quadrupole time of flight mass spectrometry (UHPLC-ESI-QTOF-MS) in negative ionization mode.

The subject of the analysis was the flowering herb and roots of micropropagated plantlets from the experimental plot (ex vitro plant material). It was chosen for phytochemical screening as easily obtainable biomass, owing to the high efficiency of micropropagation and twofold higher accumulation of ecdysteroids when compared to intact plants and in vitro systems studied earlier. Hypocotyl-derived, stabilized callus was also subjected to chemical screening [12]. The base-peak chromatograms (BPC) of ex vitro herb, ex vitro roots and callus extracts, obtained using high-resolution ultra-high-performance liquid chromatography-electrospray ionization quadrupole time of flight mass spectrometry (UHPLC-ESI-QTOF-MS), are presented in Figure 3. The detailed results of the analysis, including retention times, observed ion mass, fragmentation spectra and tentative identification are summarized in Table 1 [6,23,24,25,26,27,28,29,30].

Secondary metabolites identified in the extracts from micropropagated *L. flos-cuculi* and callus by means of UHPLC-MS analysis belong to chemical classes reported previously in the plant, namely phenolic acids, flavonoids, ecdysteroids, and triterpenoid saponins [1,6,7,12]. Other compounds have been reported for the first time in this species, including quinic acid derivatives, multiple flavonoid compounds, complex saponins of partially elucidated structure, and two ecdysteroids (Table 1). Screening of aqueous methanolic extracts revealed 26 compounds in the herb, 22 in the root and 10 in the callus. The results of our study have shown that phenolic acids were represented mainly by quinic acid derivatives and a single glycoside of benzoic acid. Flavonoids can be characterized as C-glycosyl derivatives of apigenin, luteolin, and unidentified aglycones, including compounds with additional O-glucopyranosyl or acetyl groups. Among the C-glycosyl flavonoids are vicenin II, vitexin rhamnoside and orientin derivatives. Two vitexin derivatives contain an additional O-glucopyranoside sugar residue and their C-glycosyl is acetylated at C′-6. The C-glycosyl flavonoids are unique as the aglycone is linked to the carbohydrate residue by a stable C-C bond. This leaves them unaffected by hydrolytic enzymes of the human digestive system, that usually breaks down O-glycosylated flavonoids before absorption. As a result, intact C-glycosyl flavonoids exert a spectrum of diverse activities, such as anti-diabetic, anti-inflammatory, anxiolytic, antispasmodic, and hepatoprotective [31]. Ecdysteroids included ecdysone and its diverse hydroxylated derivatives, namely 20-hydroxyecdysone (20HE), together with polypodine B (polB), viticosterone E, ajugasterone C and integristerone A. The two latter compounds are reported for the first time in the species. Triterpenoid saponins were most often the quillaic acid or gypsogenic acid glycoside derivatives, present as multiple isomers, as well as gypsogenin and oleanolic acid glycosides.

Phytochemical screening of the flowering herb and roots from ex vitro plants revealed significant differences—there were 10 flavonoid compounds in the herb, whereas none was found in roots. Flavonoids were present in roots at the stage of in vitro cultures, most probably due to the exposure to light, but it seems that the ability of roots to accumulate flavonoids was lost after transferring to ex vitro conditions. Ecdysteroids were more diverse in the roots (six different compounds), while only 20HE and polB were detected in the flowering herb. Phenolic acids in the herb were represented by quinic acid and its derivatives, while a benzoic acid derivative was found only in the root. The presence of complex triterpenoid saponins was comparably abundant in both ex vitro flowering herb (10 compounds) and roots (13 compounds), which makes them the dominant class of secondary metabolites. There were significant differences between the callus extract composition and compounds present in the herb and roots of in vitro propagated plants. Among the ten compounds detected in the callus extract, the most dominant group were seven triterpenoid saponins that were structurally similar to those in ex vitro plant material, but they were not identical. The structural complexity and diversity of saponins in this species and their biological activity requires further investigation. Polyphenols were represented by a ferulic acid derivative, an apigenin derivative, and dalpanin, an atypical C-glycosyl isoflavonoid. No ecdysteroids were found (Table 1).

### 2.4. Total Phenolic, Total Phenolic Acid and Total Flavonoid Content

Among all the investigated extracts from *L. flos-cuculi* plant materials, the inflorescence extract surpassed all the others in terms of the phenolic compound content per gram of dry weight (d.w.). The total phenolic (TP) content of the inflorescence was evaluated at 195.4 mg of GAE (gallic acid equivalent) per gram of d.w. The flowering herb of intact plants (TP for Ns-H = 112.10 mg g^−1^), the flowering herb of ex vitro plants (TP for ExV-H = 113.47 mg g^−1^) and shoots from in vitro cultures (TP for InV-S = 106.38 mg g^−1^) exhibited comparable content of total phenolics, but lower than that in the inflorescence, slightly above 100 mg g^−1^. Roots did not exceed 50 mg g^−1^ GAE of d.w., except in vitro roots, with TP content at 63.6 mg g^−1^. TP content of callus was the lowest—at 22.07 mg g^−1^. Generally, aerial parts of the plants, regardless of the origin (natural site, in vitro cultures, ex vitro plants), showed the highest content of TP. The comparison is summarized in Table 2.

The total phenolic acid (TPA) content and the total flavonoid (TF) content indicated many similarities. These parameters were both the highest in inflorescences (TPA for Ns-F = 3.88 mg g^−1^; TF for Ns-F = 1469 µg g^−1^). The herb of a plant from the natural site had a higher content of those compounds (TPA for Ns-H = 2.62 mg g^−1^; TF for Ns-H = 1232 µg g^−1^) than the herb of ex vitro plant (TPA for ExV-H 2.25 mg g^−1^; TF for 889 µg g^−1^), both exceeding in vitro shoot culture material, where shoots contained almost 50% more phenolic acids and flavonoids than roots. The most significant differences were the high phenolic acid content in callus (2.13 mg g^−1^), nearly as high as in the flowering herb (2.25 mg g^−1^), and the absence of flavonoids in callus and roots. The total phenolic acid content of roots from both ex vitro and the natural site plants was similarly low, at about 0.5 mg g^−1^. The exception was the presence of flavonoids in adventitious roots of in vitro plantlets cultured under photoperiod conditions, which were the only roots containing flavonoids in this species. The data are presented in Table 2.

### 2.5. Preliminary Determination of Antioxidant Activity and Radical Scavenging Activity

Both assays employed, i.e., ferric reducing antioxidant potential (FRAP) and 2,2-diphenyl-1-picrylhydrazyl radical scavenging activity (DPPH), revealed that the material possessing the strongest antioxidant activity was the inflorescence, consequently followed by flowering herbs.

FRAP assay results demonstrated very similar activity of the extracts from herbs collected from plants growing in the natural site (intact) and in the experimental plot (ex vitro), at 11.46 and 11.42 mg of ascorbic acid equivalent (AAE) g^−1^ d.w., respectively. The extracts of shoots from in vitro cultures and all types of roots show similar potency (ranging from 5.13 to 5.96 mg AAE g^−1^ d.w.). Callus exhibited the weakest activity, barely reaching 2.02 mg AAE g^−1^ d.w. The results are shown in Table 2.

The results of the DPPH assay were more diversified and the differences between the investigated materials were more pronounced. There was a significant difference between the natural site herb and micropropagated herb, with the latter being approximately half as active as the former. In vitro shoots and roots showed comparable activity, followed by the natural site root and ex vitro root as the weakest extract in terms of DPPH radical reduction. The callus extract at the highest concentration studied barely reached IC_25_. The results are presented in Table 2.

### 2.6. The Correlation between Phenolic Content and Antioxidant Activity

The results indicate a moderately high correlation between total phenolic content (TP) and antioxidant activity, based on linear regression coefficients (FRAP, R^2^ = 0.89; DPPH, R^2^ = 0.66). For TPA content, the correlation is lower, affected by the fairly high phenolic acid content of callus but weak antioxidant activity (FRAP, R^2^ = 0.63; DPPH, R^2^ = 0.78). For TF content, it is considerably high, (FRAP, R^2^ = 0.78; DPPH, R^2^ = 0.83). These results suggest that although phenolic acids and flavonoids are mostly responsible for the antioxidant activity observed, the extracts may contain compounds of different structure that contribute to it.

## 3. Discussion

The studied species has been recently introduced to in vitro cultures by our team, focusing on establishing the efficient micropropagation protocol and developing various in vitro systems with the ability to synthesize ecdysteroids. The content of main ecdysteroids (20HE, polB) was determined in the diverse plant material—intact plants, in vitro cultures and ex vitro plants, with the highest levels found in the flowering herb of micropropagated plants from the experimental plot (ex vitro plants) [12].

Ethnomedicinal uses of *Lychnis flos-cuculi* and the related species from this family were summarized by Chandra and Rawat [3]. Ragged Robin flowers were reported to be used as a decoction for treatment of headache, malaria and stomach pains. Other *Lychnis* species include *L. coronaria*, used for treating leprosy and diarrhea, healing wounds including inflammation, whereas its roots were confirmed to show the hepatoprotective activity. Another species, *L. coronata*, was used for the treatment of skin infections and inflammation, including herpes. Closely related *Silene* species are used for curing sore throat, treatment of fever, as a remedy for various inflammations, and to prevent the common cold. Moreover, they are used against hair infestation, as an ophthalmic medicine, and also for rinsing wounds and washing hair [3].

In light of the described ethnomedicinal use it is reasonable and justified to further investigate the biological activity of *L. flos-cuculi*, as the known studies concerning this subject only mention aerial parts of the plant and their antibacterial and antioxidant properties [7,8]. The reports describing phytochemical constituents of Ragged Robin span several decades. Completing the knowledge of the secondary metabolites produced by the plant, with the aid of modern analytical methods and use of alternative biotechnological sources, can help design the future research of biological activity.

Regarding phytochemical screening of both aerial parts and roots of ex vitro plants at the stage of flowering, there are certain discrepancies between the literature data and the results of our analysis. Earlier reports mention simple hydroxy- and methoxy-derivatives of both benzoic and *trans*-cinnamic acids in *L. flos-cuculi* [6], while Costea et al. [7] identified ferulic and caffeic acids in aerial parts. However, neither TLC analysis nor phytochemical screening unambiguously confirmed the presence of these compounds, even in intact plants. In addition, previously unreported quinic acid derivatives, including chlorogenic acid isomers, have been reported by us in the flowering herb of plants from the experimental plot. Triterpenoid saponins, present in both aerial parts and roots, can be considered the dominant group of secondary metabolites of the species. In our study, phytochemical screening revealed a multitude of complex compounds, characterized as oleanane-type aglycones linked to diverse carbohydrate residues. As is already known from the literature, glycosides of gypsogenin, gypsogenic acid and quillaic acid are considered characteristic triterpenoid saponins of Caryophyllaceae family [32]. Moreover, not all ecdysteroids reported earlier by Bathori et al. [5] and Mamadalieva et al. [8] were detected during HPLC screening mentioned in this paper. This may be due to the trace amounts of those compounds compared to the content of main ecdysteroids and the large-scale isolation undertaken by Bathori et al. [5] in contrast to phytochemical screening presented herein, or a marginally different chemical profile of ex vitro plants compared to those from the natural site.

To the best of our knowledge, the only similar analyses of *L. flos-cuculi*, regarding the phenolic content and the antioxidant capacity, were performed by Costea et al. [7]. The sum of phenolics was determined for the flowering herb, which was also tested by our team, however, the values cannot be directly compared due to the different equivalent adopted. The results were also expressed per dry mass of extract, not dry weight of the raw material. The aqueous ethanolic extract (50%) from the aerial part of the intact plant was tested in terms of the total phenolic content (expressed as 5.1 g tannic acid equivalent per 100 g of the dry extract) and the flavone content (0.36 g quercetin/100 g dry extract). The latter can be compared to our result when expressed as 0.452 g QE (quercetin equivalent) per 100 g of dry extract. Costea et al. assessed the reducing power of the aqueous ethanolic extract (50%) from the aerial part by means of FRAP assay (14.8 mg AAE g^−1^ extract) and the value was lower compared to our result for the flowering herb dry extract (42.04 mg AAE g^−1^ extract). Additionally, the differences in the results of ABTS radical assay hint that the choice of the solvent used for the extraction can significantly affect the quantitative composition of the extract and should be carefully considered when comparing the results. AAE of 50% aqueous ethanolic and aqueous extracts from aerial parts were 287 and 523 mg g^−1^, respectively [7].

Several other species of the interrelated genus *Silene* were tested for phenolic compounds or the antioxidant activity. The methanolic extracts of three Iranian species *Silene gynodioca*, *S. spergulifolia* and *S. swertiifolia* (aerial parts) were tested for their TP and TF content, as well as the antioxidant activity, using DPPH assay. Compared to *L. flos-cuculi* natural site flowering herb dry extract (411.2 mg GAE g^−1^), the TP content in extracts of all three species was much lower (50.81, 47.34, 65.66 mg GAE g^−1^, respectively). The TF content in extracts was similar and slightly higher, at 5.03, 4.77 and 5.64 mg QE g^−1^, respectively, compared to our result of 4.52 mg QE g^−1^ for *L. flos-cuculi* flowering herb extract. The DPPH radical scavenging activity expressed as IC_50_ was in a range between 0.28 to 0.13 mg mL^−1^, which is much higher compared to 2.99 mg mL^−1^ [33].

Many of these studies are preliminary and reveal only a fragmentary view of total phenolic content or antioxidant activity by employing certain methods, investigating different types of extracts, often focusing on aerial parts as the only plant material, with results expressed as different equivalents and units. It is, therefore, difficult to effectively compare the quantity of phenolic constituents and antioxidant activity in certain cases.

The polyphenolic composition of calli is often low in flavonoid compounds and relatively high in phenolic acids. In the triterpenoid-compound-producing species, such as *Chaenomeles japonica*, several types of callus investigated by Kikowska et al. [34] were similarly more abundant in phenolic acids than in flavonoids. The callus line containing the least polyphenols was especially high in pentacyclic triterpenoids. Equally, the callus of *L. flos-cuculi* was exceptionally rich in triterpenoid saponins.

Phenolic acids and flavonoids are secondary metabolites most significantly contributing to the antioxidant activity of the plant material. Phenolic acids are ubiquitous in plants and are direct precursors of flavonoids. Both classes serve multiple roles, like being intermediates in the production of structural polymers such as lignin, condensed tannins, anthocyanins and aromatic amino acids. They protect plants from ultraviolet radiation, function as phytoalexins and, therefore, are accumulated in relatively large amounts. The structure of phenolic acids and flavonoids facilitates the scavenging of free radicals and reduction of metal cations, which are main mechanisms of their antioxidant activity and principle behind colorimetric assays [22].

However, no additional antioxidant activity seems to be contributed by ecdysteroids which, according to the literature data, also exert antioxidant effects [35,36,37]. It was previously reported that micropropagated plants growing in the ground (ex vitro herb and roots) contain approximately twice as much ecdysteroids, when compared to natural state organs [12], but there is no significant increase in the antioxidant activity when comparing these materials. In fact, 20HE demonstrated only mild radical scavenging activity, as shown by Miliauskas et al. [36]. It is likely that the antioxidant properties of ecdysteroids are enzyme-dependent and differ from the straightforward redox reactions of polyphenols, being rather involved in signal transduction leading to the antioxidant response in live cells [35,37]. To evaluate the additional antioxidant effect of ecdysteroids, the enzyme-dependent methods such as lipooxygenase assay used by Bathori et al. [35] should be applied.

Considering the results of total phenolics and antioxidant assays, shoot cultures and aerial parts of *L. flos-cuculi*, especially those containing flowers, can be described as having moderate to high polyphenol content, high flavonoid content and moderate antioxidant activity. Roots and callus are moderately low in polyphenols (about two and four times less than the aerial parts, respectively), contain, at most, traces of flavonoids and demonstrate low antioxidant activity (about two times lower than aerial parts). While in-vitro-derived roots are an exception, accumulating moderate levels of flavonoids, this does not seem to significantly contribute to their antioxidant capacity.

On the basis of the literature on the subject [1,6,7] and our results, the aerial parts of the species are a substantial source of polyphenols, mainly flavonoids. The mature micropropagated plants are also an especially abundant source of ecdysteroids [12], known for their wound-healing and burn-healing properties. Therefore, the extracts from *L. flos-cuculi* might be used topically as a dermatological preparation. However, at the stage of the current studies, as the biological activity of saponin constituents remains unknown, it is still too early to safely consider the use of the plant or its preparations.

## 4. Materials and Methods

### 4.1. Plant Material and In Vitro Cultures

Flowering plants and mature seeds of *L. flos-cuculi* were collected from a meadow nearby Kuźnica Trzcińska, Wielkopolskie Voivodeship, Poland (51°09′21′′ N 18°03′24′′ E) in June 2016. The voucher specimens (No. CP-Lfc-2016-0601) and seeds (No. CP-Lfc-2016-0602) were deposited in the Herbarium of Department of Pharmaceutical Botany and Plant Biotechnology of Poznan University of Medical Sciences. Likewise, the micropropagated flowering plants from experimental plot (52°24′12′′ N, 16°56′25′′ E) were gathered in June 2018 and deposited as voucher specimens (No. CP-Lfc-2018-0601) along with the seeds (No. CP-Lfc-2018-0602). The protocols for efficient micropropagation through axillary bud formation and the development of various in vitro systems of *L. flos-cuculi* were described in our previous article [12]. Multiplied shoots are homogenous plant material with stable genome size, which was confirmed in earlier studies. For the presented experiment, a sufficient number of shoots was multiplicated through a series of subcultures according to this protocol (Figure 1a–c). Acclimatized plantlets were transferred to the experimental plot (ex vitro plants, Figure 1f), where they developed an abundant root system, flowers and fruits. Hypocotyl-derived callus was cultured on MS (Murashige and Skoog, [38]) medium containing 2,4-dichlorophenoxyacetic acid (1.0 mg L^−1^ 2,4-D) and N^6^-furfuryladenine (0.1 mg L^−1^ kinetin). After six subcultures, uniform bright and soft callus was collected.

All in vitro cultures were kept in phytotron, equipped with fluorescent lamps emitting cool-white light of 55 μmol m^−2^ s^−1^ intensity, under 16:8 h photoperiod at temperature of 21 ± 2 °C, except callus cultures, which were kept in the dark.

### 4.2. Preliminary TLC Analysis

The dried and pulverized plant material (3.0 g) was extracted three times with 80% aqueous methanol (30 mL) under reflux and the resulting extracts were dried under a rotary evaporator. The extracts were subjected to thin-layer chromatography (TLC) analysis for the presence of phenolic compounds. The samples were applied on 20 × 10 cm^2^ silica gel plate (Merck, Darmstadt, Germany), which was developed in a mobile phase consisting of ethyl acetate-acetic acid-water (8:1:1, *v/v/v*) and sprayed with 0.1% NA (aminoethyl diphenylborate) ethanolic solution to visualize flavonoids and phenolic acids. The plates were inspected under UV light (366 nm). Additionally, the callus extract was applied on 10 × 10 cm^2^ cellulose plate (Merck, Darmstadt, Germany) and the two-dimensional chromatogram (2D-TLC) was developed. The first mobile phase consisted of *n*-butanol-acetic acid-water (4:1:5), while 15% aqueous solution of acetic acid was used as the second mobile phase. After development, the plate was sprayed with 1% ethanolic AlCl_3_ solution and inspected under UV light (254 nm).

### 4.3. UHPLC-MS Analysis

Reagents: methanol and acetonitrile, HPLC grade, were purchased from Merck (Darmstadt, Germany). Formic acid, LC-MS grade, was purchased from Sigma-Aldrich, (St. Louis, MO, USA). Ultrapure water was obtained in-house with a purification system (Milli-Q-Simplicity-185, Millipore Corp., Darmstadt, Germany).

#### 4.3.1. Plant Material and Preparation of Samples

Dried aerial parts and roots of four-month-old plants and lyophyllized callus (100 mg) were extracted using an automatic extractor Dionex ASE 200. The material was extracted with 80% aqueous methanol at 40 °C. The extracts were evaporated to dryness under reduced pressure at 40 °C (a rotary evaporator, Heidolph Hei-Vap Advantage, Schwabach, Germany). UHPLC samples were dissolved in 2 mL of 80% methanol and sonicated, reaching the final concentration of 50 mg mL^−1^.

#### 4.3.2. UHPLC Conditions

The qualitative analysis of herb, root and callus extracts was carried out using ACQUITY UPLC system, equipped with PDA and a triple quadrupole mass detector (TQD, Waters, Milford, MA, USA). Chromatographic separation was performed using Acquity UPLC BEH C18 (100 × 2.1 mm^2^, 1.7 µm particle size; Waters, Manchester, UK), and column temperature was maintained at 40 °C. The mobile phases were acidified (0.1% formic acid) water (solvent A) and acidified (0.1% formic acid) acetonitrile (solvent B), and the chromatographic method utilized the following linear gradient: from 5% B to 50% B over 24 min. The sample injection volume was 3.5 µL, and the flow rate was set at 0.4 mL min^−1^.

The compounds were analyzed on the basis of the data from mass spectra. ESI ionization was performed in negative ion mode. Ions’ source parameters were as follows: capillary voltage—2.8–3.1 kV, con voltage—45.0 V, and source and desolvation temperatures were maintained at 137 °C and 350 °C, respectively. The flow of collision gas was used as cone at 100 L h^−1^, and the flow of desolvation gas at 800 L h^−1^. The collision gas flow was 0.1 mL min^−1^. In addition, phenolic compounds were analyzed on the basis of the data from UV spectrum. PDA was operated in the range of 191–489 nm, with the resolution of 3.6 nm. Data processing was performed using MassLynx V4.1 software, Waters.

### 4.4. Determination of the Total Phenolic (TP), Total Phenolic Acid (TPA) and Total Flavonoid (TF) Content

The ground and dried plant material (5.0 g) was extracted three times with the matched volume (50 mL) of 70% aqueous methanol for 1 h under reflux at 80 °C. After evaporation to dryness, the extract was redissolved in 50 mL of water (equivalent to 0.1 g of the dry plant material per 1 mL) and frozen. A range of aqueous solutions of the extracts (dilutions ranging between 1:50 and undiluted solution) was tested during the following colorimetric assays.

For determination of the total phenolic content in the tested extracts, the modified Folin–Ciocalteau reagent method was applied [39] with gallic acid as an external standard to plot the calibration curve. The results were expressed as milligrams of gallic acid equivalent (GAE) per gram of dry weight of the plant material.

For evaluation of the total phenolic acid content in the tested samples, Arnov’s reagent spectrophotometric method was applied according to Polish Pharmacopoeia VI edition [40]. Caffeic acid was used as an external standard to plot the calibration curve. The results were expressed as milligrams of caffeic acid equivalent (CAE) per gram of dry weight of the plant material.

The total flavonoid content assay was performed as described by Meda et al. [41], with quercetin as an external standard to plot the calibration curve. The results were expressed as micrograms of quercetin equivalent (QE) per gram of dry weight of the plant material.

All of the above assays were performed using the Multiskan GO (Thermo Fisher Scientific, Vantaa, Finland) spectrophotometer. The values were expressed as the mean of six replications ± SD.

### 4.5. Determination of the Antioxidant Capacity

To assess the antioxidant capacity of the extracts prepared as described above, FRAP (ferric reducing antioxidant potential) method was employed, according to the modified protocol of Benzie and Strain [42]. The FRAP assay was chosen to measure the potential of the samples to engage in redox reactions, in this case, the reduction of the Fe^3+^ cations complexed by aromatic heterocyclic amine. The evaluation was performed using the Lambda 35 UV/Vis Spectrophotometer (Perkin Elmer, Waltham, MA, USA). Ascorbic acid was used as an external standard to plot the calibration curve and the results of the antioxidant activity were expressed as milligrams of ascorbic acid equivalent (AAE) per gram of dry weight of the plant material. The values were expressed as the mean of six replications ±SD.

### 4.6. Determination of the Radical Scavenging Activity

To evaluate the radical scavenging activity of the aforementioned extracts, DPPH (2,2-diphenyl-1-picrylhydrazyl radical) method was utilized, based on modified protocol of Annegowda et al. [43]. The assay is based on a stable DPPH radical changing color after gaining an electron, and therefore it was selected to quantify the radical scavenging capacity of the samples. The DPPH assay was performed using the Multiskan GO (Thermo Fisher Scientific, Vantaa, Finland) spectrophotometer. DPPH radical scavenging ability was calculated using the following equation:

*DPPH radical scavenging* [%] = (*A0* − *Ax*)/*A0* × 100% (1)

where *A0*—absorbance of negative control, *Ax*—absorbance of the sample. IC_50_ values of the respective samples were calculated as the concentration required to scavenge 50% of DPPH radical. The concentration was converted to grams of the dry plant material per mL. The values were expressed as the mean of six replications ±SD.

### 4.7. Statistical Analysis

The obtained data were analyzed using a one-way analysis of variance (ANOVA) and the statistical significance was determined by Duncan’s POST-HOC test (*p*-value of 0.05). All the analyses were conducted using STATISTICA v.13 (StatSoft, Inc., Tulsa, OK, USA, 2015).

## 5. Conclusions

Among the tested plant materials, the inflorescence of *L. flos-cuculi* is the richest in phenolic acids and flavonoids, as well as exhibits the highest antioxidant activity, followed by materials containing flowers, that is, the flowering herb of either origin. The results suggest that the antioxidant activity measured by colorimetric assays is related to the polyphenol content and unaffected by high ecdysteroid accumulation. To our knowledge, this has been the first investigation of the total polyphenol content and the antioxidant activity of *L. flos-cuculi* in vitro propagated plants, including their underground parts. The results of phytochemical screening prove that micropropagated Ragged Robin produces secondary metabolites as in intact plants. It can constitute an alternative source of valuable, homogenous plant biomass, fit for further research into phytochemical composition and biological activities.

## Figures and Tables

**Table 1:** Secondary metabolites identified in the extracts from *Lychnis flos-cuculi* callus and ex vitro flowering herb and roots with the use of ultra-high-performance liquid chromatography mass spectrometry (UHPLC-MS).

No.	Rt (min)	Tentative Identification	UV (nm)	[M + H]^+^ m/z	[M − H]^−^ m/z	MS2	Compound Class	Present in:	References
1	0.66	quinic acid derivative	197		191	377, 191	PA	R	not found
2	0.68	quinic acid derivative	197		377	377, 191	PA	H	not found
3	0.87	quinic acid	195		191	191	PA	H, R	not found
4	2.46	quinic acid derivative			355	355, 191	PA	H	not found
5	3.01	quinic acid derivative			355	355, 209, 191	PA	H	not found
6	4.8	ferulic acid derivative	198, 282		195	136, 195	PA	C	not found
7	5.04	apigenin derivative	218, 290, 350	433		253, 271, 433	F	C	not found
8	5.39	benzoic acid glycoside	198, 224		253	253, 121	PA	R	not found
9	6.05	dalpanin	202, 222, 327		533	267, 353, 533	F	C	not found
10	6.5	apigenin-6,8-di-C-β-D-glucopyranoside (vicenin II)	212, 269, 350	595		595, 449, 431, 329	F	H	[6,21]
11	7.48	vitexin rhamnoside (vitexin derivative)	214, 270, 339	579		579, 433, 415, 313	F	H	[6,21]
12	8	luteolin-8-C-β-D-glucopyranoside derivative (orientin derivative)	215, 269, 341		619	619, 607, 447, 323	F	H	[6,21]
13	8.14	apigenin-5-O-β-D-glucopyranosyl, 8-C-(6″acetyl)-β-D-glucopyranoside	213, 269, 350		635	635, 619, 607, 329	F	H	[6,21]
14	8.42	20-hydroxyecdysone	247		479	525, 479	E	H, R	[22,23,24]
15	8.47	polypodine B	228		495	541, 495	E	H, R	[22,23]
16	8.83	20-hydroxyecdysone derivative (ajugasterone C)	220		479	525, 479	E	R	[22,23,24]
17	8.91	integristerone A	220		495	541, 495	E	R	[22,23,24]
18	9.16	luteolin-8-C-β-D-glucopyranoside derivative (orientin derivative) isomer I	214, 270, 338		619	619, 577, 495, 315	F	H	[6,21]
19	9.51	unknown C-glycosyl derivative	215, 270, 339		661	661, 619, 523	F	H	[21]
20	9.7	unknown C-glycosyl derivative	215, 271, 330		705	705, 661, 619, 309	F	H	[21]
21	9.85	quillaic acid or gypsogenic acid-triterpene glycoside derivative			972	972, 648, 485, 323	TS	R	[25]
22	10.09	ecdysone		465		465, 447, 429	E	R	[24,26,27]
23	10.6	apigenin-5-O-β-D-glucopyranosyl, 8-C-(6″acetyl)-β-D-glucopyranoside derivative	219, 271, 330	621		621, 475, 379, 313	F	H	[6,21]
24	10.76	viticosterone E	222		521	567, 521	E	R	[27,28]
25	10.83	unknown C-glycosyl derivative	216, 270, 338		661	661, 619, 509	F	H	[21]
26	11.34	unknown C-glycosyl derivative	217, 270, 338		703	747, 703, 661	F	H	[6,21]
27	11.81	quillaic acid or gypsogenic acid-triterpene glycoside derivative		649		649, 487, 469, 325	TS	R	[25]
28	12.13	quillaic acid or gypsogenic acid-triterpene glycoside derivative		730		730, 649, 487, 325	TS	R	[25]
29	12.53	quillaic acid or gypsogenic acid-triterpene glycoside derivative			1150	1150, 663, 485, 351	TS	H	[25]
30	12.68	quillaic acid or gypsogenic acid-triterpene glycoside derivative			735	735, 648, 485, 323	TS	R	[25]
31	12.95	quillaic acid or gypsogenic acid-triterpene glycoside derivative, isomer I			1134	1134, 647, 485, 310	TS	H	[25]
32	13.88	quillaic acid or gypsogenic acid-triterpene glycoside derivative			1134	1134, 711, 647, 485, 323	TS	R	[25]
33	13.89	quillaic acid or gypsogenic acid-triterpene glycoside derivative, isomer II			1134	1134, 647, 485, 310	TS	H	[25]
34	14.4	unidentified	not detected		659	211, 329, 659	TS	C	not found
35	14.61	gypsogenin-triterpene glycoside derivative			1231	639, 469, 350	TS	H	[25]
36	14.62	gypsogenin-triterpene glycoside derivative			1231	638, 469, 307	TS	R	[25]
37	15.3	quillaic acid or gypsogenic acid-triterpene glycoside derivative	not detected		880	191, 405, 485, 761, 880	TS	C	not found
38	15.48	quillaic acid or gypsogenic acid-triterpene glycoside derivative, isomer III			972	972, 485, 312	TS	H	[25]
39	15.8	quillaic acid or gypsogenic acid-triterpene glycoside derivative	not detected		799	191, 330, 405, 485, 661, 799	TS	C	not found
40	16.14	unidentified			1116	1116, 771, 329	TS	H	not found
41	16.49	unidentified			1684	842, 792, 624, 329	TS	H	not found
42	16.54	quillaic acid or gypsogenic acid-triterpene glycoside derivative	not detected		901	191, 405, 485, 761, 901	TS	C	[25]
43	16.61	quillaic acid or gypsogenic acid-triterpene glycoside derivative	not detected		769	405, 411, 485, 761, 769	TS	C	not found
44	16.94	oleanolic acid-triterpene glycoside derivative			1452	726, 467	TS	R	[25]
45	16.97	quillaic acid or gypsogenic acid-triterpene glycoside derivative	not detected		820	191, 330, 405, 485, 661, 820	TS	C	not found
46	17.41	quillaic acid or gypsogenic acid-triterpene glycoside derivative		1138		726, 487, 469, 189	TS	R	[25]
47	17.63	unidentified			929	929, 883	TS	H	not found
48	18.39	unidentified			1364	682	TS	H	not found
49	18.79	quillaic acid or gypsogenic acid-triterpene glycoside derivative		1494		749, 487, 393, 189	TS	R	[25]
50	19.06	gypsogenin-triterpene glycoside derivative			717	717, 469, 453, 189	TS	R	[25]
51	19.2	quillaic acid or gypsogenic acid-triterpene glycoside derivative	not detected		1275	177, 405, 485, 599, 660, 1275	TS	C	not found
52	19.5	unidentified			1406	703	TS	H	not found
53	19.75	quillaic acid or gypsogenic acid-triterpene glycoside derivative, isomer I			768	768, 485, 435, 323	TS	R	[25]
54	20.02	quillaic acid or gypsogenic acid-triterpene glycoside derivative, isomer II			768	768, 485, 435, 323	TS	R	[25]
55	20.37	gypsogenin-triterpene glycoside derivative			739	739, 469, 453, 189	TS	R	[25]

Abbreviations: Rt—retention time; UV—absorbance peak maxima; [M + H]^+^, [M − H]^−^—pseudomolecular ion masses; MS2—fragmentation masses; Compound classes: PA—phenolic acids, F—flavonoids, E—ecdysteroids, TS—triterpenoid saponins; Present in: C—callus, H—ex vitro flowering herb extract, R—ex vitro root extract. Underlined masses represent the most intense ion.

**Table 2:** Results of spectrophotometric analysis of total polyphenol content in *Lychnis flos-cuculi* dry plant material and its antioxidant activity. The values were expressed as the mean of six replications ±SD. Mean values within each column with the same letter are not significantly different at *p* = 0.05 using Duncan’s Multiple Range test.

Plant Material	Total Phenolics (mg GAE g^−1^ d.w.)	Total Phenolic Acids (mg CAE g^−1^ d.w.)	Total Flavonoids (µg QE g^−1^ d.w.)	FRAP (mg AAE g^−1^ d.w.)	DPPH, IC_50_ (mg d.w. mL^−1^)
Ns-F	195.40 ± 4.68 ^a^	3.88 ± 0.22 ^a^	1469 ± 76 ^a^	20.14 ± 0.62 ^a^	4.33 ± 1.51 ^a^
Ns-H	112.10 ± 6.77 ^b^	2.62 ± 0.14 ^b^	1232 ± 55 ^b^	11.46 ± 0.16 ^b^	10.97 ± 0.33 ^b^
Ns-R	41.86 ± 1.31 ^d^	0.52 ± 0.03 ^f^	14 ± 3 ^f^	5.72 ± 0.37 ^c^	52.78 ± 3.03 ^e^
InV-S	106.38 ± 5.91 ^b^	1.60 ± 0.05 ^d^	701 ± 50 ^d^	5.96 ± 0.23 ^c^	43.47 ± 1.77 ^d^
InV-R	63.60 ± 6.31 ^c^	1.08 ± 0.27 ^e^	470 ± 23 ^e^	5.13 ± 0.13 ^d^	44.22 ± 2.24 ^d^
InV-C	22.07 ± 0.68 ^e^	2.13 ± 0.17 ^c^	0 ± 0 ^g^	2.02 ± 0.13 ^e^	>100 ^1^
ExV-H	113.47 ± 4.95 ^b^	2.25 ± 0.09 ^c^	886 ± 49 ^c^	11.42 ± 0.63 ^b^	19.58 ± 0.75 ^c^
ExV-R	43.54 ± 5.30 ^d^	0.44 ± 0.05 ^g^	7 ± 3 ^f^	5.18 ± 0.41 ^d^	93.30 ± 3.99 ^f^

Abbreviations: inflorescences (Ns-F), flowering herb (Ns-H), and roots of plants gathered from natural site (Ns-R); in vitro shoot cultures (InV-S), in vitro-regenerated adventitious roots (InV-R), and callus tissue (InV-C); flowering herb (ExV-H) and roots of ex vitro plants (ExV-R); GAE—gallic acid equivalent, CAE—caffeic acid equivalent, QE—quercetin equivalent; FRAP—ferric reducing antioxidant potential, AAE—ascorbic acid equivalent, DPPH—2,2-diphenyl-1-picrylhydrazyl radical; d.w.—dry weight. ^1^ the highest concentration of callus extract studied (0.1 g d.w. mL^−1^) barely reached 25% of DPPH inhibition.

## Supplementary Materials

The following are available online at https://www.mdpi.com/2223-7747/10/2/206/s1, Figure S1: 2D-TLC chromatogram of *Lychnis flos-cuculi* callus 70% aqueous methanolic extract.
