# Supplementary material for: Phytochemical Screening, Phenolic Compounds and Antioxidant Activity of Biomass from Lychnis flos-cuculi L. In Vitro Cultures and Intact Plants
Source: Plants (Basel). 2021 Jan 22;10(2):206. doi: 10.3390/plants10020206 (PMC7911596; doi:10.3390/plants10020206)
Supplement: Supplementary file 1 [file plants-10-00206-s001.pdf]

Supplementary Material

Article

Phytochemical Screening, Phenolic Compounds and Antioxidant Activity of Biomass from *Lychnis flos-cuculi* L. In Vitro Cultures and Intact Plants

Michał P. Maliński [1,*], Małgorzata Anna Kikowska[1], Agata Soluch[2], Mariusz Kowalczyk[2], Anna Stochmal[2] and Barbara Thiem[1]

1 Chair and Department of Pharmaceutical Botany and Plant Biotechnology, Poznan University of Medical Sciences, 14 Św. Marii Magdaleny St., 61-861 Poznań, Poland; kikowska@ump.edu.pl (M.A.K.); bthiem@ump.edu.pl (B.T.)

2 Department of Biochemistry and Crop Quality, Institute of Soil Science and Plant Cultivation, State Research Institute, 8 Czartoryskich St., 24-100 Puławy, Poland; asoluch@iung.pulawy.pl (A.Sol.); mkowalczyk@iung.pulawy.pl (M.K.); asf@iung.pulawy.pl (A.S.)

* Correspondence: mpmalinski@ump.edu.pl

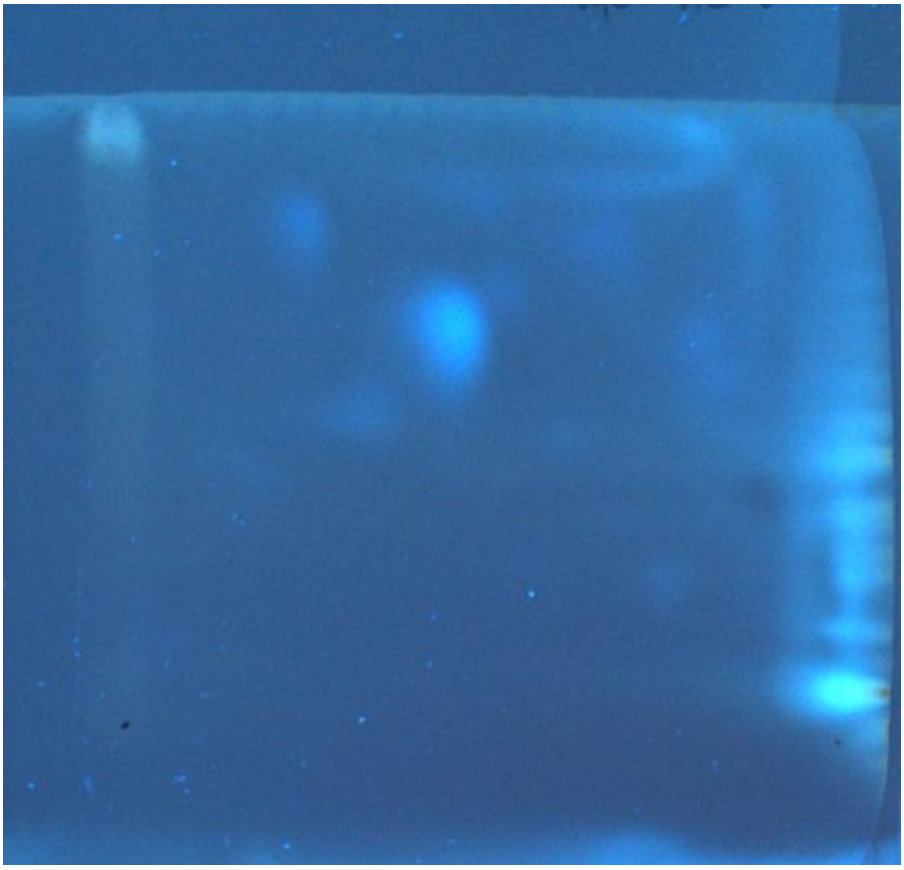

Figure S1. 2D-TLC chromatogram of *Lychnis flos-cuculi* callus 70% aqueous methanolic extract. Stationary phase: cellulose. Mobile phases: vertical: butanol-acetic acid-water (4:1:5), horizontal: 15% acetic acid. Observed under 254 nm UV light after derivatization with 1% ethanolic aluminium chloride solution.
